# Influence of chlamydia infection history on tubal patency: A pilot study

**DOI:** 10.5935/1518-0557.20260036

**Published:** 2026

**Authors:** Julià Alvarez-Puig, Nuria Muñiz-López, Laura Melé, Montserrat Cubo-Abert, Alba Noguera-Solà, Nuria Esteve-Vila, José Moreno-Sepulveda, Ana Calvo-Redol, Marta Simó-Gonzalez, Juan J Espinós

**Affiliations:** 1 Department of Obstetrics and Gynecology and IRB (Institut de Recerca Biomèdica), Arnau de Vilanova Universitary Hospital, Universitat de Lleida, Lleida, Spain; 2 Obstetrics and Gynecology Department, Autonoma University of Barcelona (UAB), Campus of Bellaterra, Cerdanyola del Vallès, Spain; 3 Clínica de La Mujer Medicina Reproductiva, Viña del Mar, Chile; 4 Fertty Clinic, Fertty Foundation, Barcelona, Spain

**Keywords:** tubal patency, hysterosalpingo-foam-sonography, Chlamydia, infertility

## Abstract

**Objectives:**

*Chlamydia* infection is a known risk factor for tubal occlusion and subsequent infertility. A significant proportion of *Chlamydia* infections are asymptomatic, which can delay diagnosis and allow silent progression of tubal damage. Therefore, identifying a history of *Chlamydia* infection-even in the absence of symptoms-may help recognize women at risk of tubal involvement. In such cases, it would be justified to perform tubal patency testing to assess potential tubal and pelvic sequelae. Our purpose was to evaluate the relationship of *Chlamydia* IgG serology with tubal patency as determined by Hysterosonosalpingo-Foam-Sonography (HyFoSy). Furthermore, the study examines whether this information could guide decision-making in the initial clinical evaluation of infertile patients.

**Methods:**

A retrospective pilot study was performed including all patients referred for primary infertility to the Reproductive Medicine Unit of the Gynecology Service of the Hospital Arnau de Vilanova in Lleida between 2022 and 2024. *Chlamydia* infection history was assessed through serological IgG testing, and tubal patency was evaluated using HyFoSy.

**Results:**

Seventy-nine patients were included in the study. A *Chlamydia* IgG seropositivity rate of 13.9% was observed, while tubal occlusion-assessed through HyFoSy-was detected in 25.3% of cases. Patients younger than 33 years with a positive *Chlamydia* IgG testing showed a higher rate of tubal occlusion compared to patients with negative serology (OR 8.00, 95% CI [1.08, 59.16]). No differences were observed in older patients.

**Conclusions:**

*Chlamydia* infection may be associated with a higher risk of tubal occlusion in younger patients, especially those under 33 years.

## INTRODUCTION

*Chlamydia trachomatis* infection is a widespread sexually transmitted infection, especially among young, sexually active individuals, and represents a significant global public health issue. In 2020, an estimated 128.5 million new cases of *Chlamydia* were recorded among individuals aged 15-49, with a 4% prevalence among women (World Health Organization, 2023). Two-thirds of these infections occurred in individuals aged 15-24. Additionally, a substantial number of asymptomatic carriers remain undiagnosed and therefore do not seek testing (Centers for Disease Control and Prevention, 2024). *Chlamydia* infection can be cured with antibiotic treatment. Patients with clinical or subclinical infection who do not undergo antibiotic treatment may develop serious health problems such as pelvic inflammatory disease (PID). The consequences of PID include irreversible impairment of tubal function and patency, hydrosalpinx, ectopic pregnancy, and/or chronic pelvic pain (World Health Organization, 2023; Centers for Disease Control and Prevention, 2024).

There appears to be a possible association between *Chlamydia* IgG seropositivity and tubal infertility, with a reported prevalence of up to 20% among asymptomatic infertile women (Morhason-Bello *et al*., 2014). Therefore, this test might represent a useful diagnostic tool in the infertility work-up, potentially aiding in the identification of women at increased risk of tubal involvement who could benefit from further tubal and pelvic assessment (Lim *et al*., 2011). The test, which detects IgG antibodies in serum as an indicator of past or persistent *Chlamydia* infection, is being considered for incorporation into routine screening of infertile couples prior to initiating assisted reproductive technologies (ART). It is easy, quick, inexpensive, non-invasive, and can be performed at any point in the menstrual cycle, with reasonably good sensitivity and specificity. A positive result may suggest prior, ongoing, or chronic infection (Fiddelers *et al*., 2005; Morhason-Bello *et al*., 2014).

Tubal factor accounts for approximately 20-35% of infertility cases, including both obstructive and non-obstructive tubal dysfunction. Among these, tubal obstruction-detectable through imaging techniques-is found in 10-15% of patients undergoing infertility evaluation (Dessole *et al*., 2003; Morhason-Bello *et al.,* 2014; Levaillant *et al*., 2022). In many cases of asymptomatic tubal disease, a previous clinical or silent pelvic inflammatory episode is suspected, with *Chlamydia* trachomatis being the most frequently implicated pathogen.

The gold standard to evaluate tubal patency is still laparoscopic assessment with tubal chromoperturbation, but it is not currently considered a first-line test due to its invasiveness and cost. The classic technique for the study of tubal patency has been hysterosalpingography with iodine contrast (HSG). Hysterosalpingo-contrast sonography (HyCoSy) presents high sensitivity and specificity for the determination of tubal patency, showing good association with HSG results (Dessole *et al.*, 2003). Hysterosonosalpingo-Foam-Sonography (HyFoSy) is used when the contrast medium used is foam, significantly improving diagnostic accuracy and efficacy compared to HyCoSy, being equal or superior in sensitivity and specificity (Exalto & Emanuel, 2019; Lim *et al.*, 2015).

At present, there are no published studies associating HyCoSy or HyFoSy, tests that assess tubal patency non-invasively, with a history of *Chlamydia* infection, one of the main causes of tubal involvement. To date, no study has specifically evaluated the correlation between HyFoSy findings and Chlamydia trachomatis IgG serology, which represents a novel aspect of the present research.

The aim of this pilot study is to evaluate the association between *Chlamydia* IgG serology and tubal patency as assessed by HyFoSy, as well as the tolerance and safety of HyFoSy.

## MATERIAL AND METHODS

### Study design

A retrospective pilot study conducted at the Assisted Reproduction Unit (ARU) of the Arnau de Vilanova University Hospital in Lleida between May 2022 and May 2024.

At the initial (baseline) visit, the following variables were recorded: duration of infertility, age, history of ovarian pathology or PID, body mass index (BMI), smoking status, anti-Müllerian hormone (AMH) levels and antral follicle count. In addition, *Chlamydia* trachomatis IgG serology, HyFoSy for assessment of tubal patency (unilateral or bilateral occlusion), and pain evaluation using the visual analogue scale (VAS) during and immediately after the procedure were performed. The VAS is a unidimensional tool used to assess pain intensity, allowing for the monitoring of a patient’s pain over time or comparison of pain levels between individuals with similar clinical conditions.

### Participants

The study included all female patients aged 18 to 40 years who were referred to the ARU with a diagnosis of primary infertility or a solitary desire for motherhood, and who underwent HyFoSy and *Chlamydia* trachomatis IgG serological testing. All eligible patients meeting the inclusion criteria during the study period were consecutively included. Patients who did not meet the inclusion criteria or who were ineligible for assisted reproductive techniques under the regulations of the Catalan public health system were excluded. As this was a pilot study, no formal sample size calculation was performed.

### *Chlamydia* IgG Serological Test

Serological detection of *Chlamydia trachomatis* IgG antibodies was performed using a commercial chemiluminescent enzyme immunoassay (VirClia® Chlamydia trachomatis IgG Monotest, Vircell S.L., Granada, Spain). According to the manufacturer, the assay demonstrates a sensitivity and specificity of 100%. The test detects IgG antibodies indicative of prior or recent infection.

The assay showed intra-assay variation coefficients 7%-10%, and inter-assay 8%-16%. The manufacturer’s instructions were followed for interpretation of results; the cut-off value (Index ≥ 1.1) was used to classify samples as positive, as established in the test protocol.

### HyFoSy Test Procedure

HyFoSy was performed using a Voluson S10 ultrasound system with a volumetric transvaginal probe (RIC5-9-D), following intrauterine instillation of ExEm® Foam (IQ Medical Ventures, The Netherlands). The procedure was conducted and interpreted by two expert gynecologists. Tubal patency was assessed using HyFoSy with ExEm® Foam (IQ Medical Ventures, Zuidhorn, The Netherlands). The passage of the foam was visualized as a hyperechogenic line with an anterograde path and, anatomically, it represents a trilaminar tube (black/white/black) that would be equivalent to the tube walls in black and the lumen in white. Cases with bilateral tubal patency were classified as having normal tubal status, while any unilateral or bilateral obstruction was categorized as tubal occlusion.

Peritoneal adhesions were not evaluated as part of the study protocol. Although HyFoSy may occasionally suggest the presence of adhesions through indirect ultrasonographic signs, these findings were not systematically recorded or analysed. Consequently, adhesion assessment was not included among the study variables.

### Data analysis

A univariate descriptive analysis was performed. Mean and standard deviation were calculated for quantitative variables. Shapiro-Wilk and Kolmogorv-Smirnov normality tests were applied to check the normality of these variables. For qualitative variables, the percentage of each of the categories were calculated.

A descriptive bivariate analysis was subsequently conducted based on the presence or absence of tubal patency as determined by HyFoSy. The possible relationship of the presence or absence of tubal patency with each of the study variables was assessed. For this purpose, the parametric T-test or the non-parametric Mann-Whitney test was used to compare the mean values between the two groups of the quantitative variables. For qualitative variables, comparisons between groups were made using Pearson’s Chi-square test or Fisher’s exact test, depending on the expected cell frequencies.

A multivariate logistic regression model was fitted, with tubal patency as the dependent variable and presence of *Chlamydia*-positive serology as the main independent variable. The odds ratio (OR) and its 95% confidence interval were obtained. Finally, patients were stratified by age, and fitted the same multivariate logistic regression model. No formal power calculation was performed; therefore, the statistical power to detect the primary association (between Chlamydia IgG seropositivity and tubal occlusion) was limited.

All data analysis was performed with the statistical software Stata version 16.0 and Grphpad Prism version 6.0. For all tests a statistical significance level of 5% (α = 0.05) was considered.

### Ethical approval

The present study was reviewed and approved by the Research Ethics Committee on Medicinal Products of University Hospital Arnau de Vilanova, under No. CEIC-2999. All patients were provided with the informed consent form for HyFoSy, as well as the study information document and the informed consent form for participation in the study that was completed for each study participant.

In compliance with Organic Law 3/2018, of 5 December, on Personal Data Protection and guarantee of digital rights and Regulation (EU) 2016/679 of the European Parliament and of the Council of 27 April 2016, the clinical data were recorded in a password-protected Excel database, where the identity of the patients was coded by assigning a case number, in order to preserve their identity. The case number-patient name relationship was recorded in a separate electronic document, coded and accessible only to the study PI and collaborating researchers.

## RESULTS

Seventy-nine patients accomplished the inclusion criteria and participated in the study. The mean age was 33.3 years, and the mean BMI of 25.3 kg/m^2^ (Table 1). A *Chlamydia* IgG seropositivity rate of 13.9% was observed. Tubal occlusion was identified in 25.3% of patients, including 19.0% with unilateral obstruction and 6.3% with bilateral occlusion (Table 1). Pain perception during the HyFoSy procedure, assessed using the VAS scale, was 5.2 intra-procedurally and 2.6 post-procedurally (Table 1). No procedural complications were reported. The general characteristics of the study population are presented in Table 1.

**Table 1:** Characteristics of the study sample

	N=79	%
Age (years), Mean (SD)	33.3 (3.6)	-
BMI (kg/m^2^), Mean (SD)	25.3 (4.9)	-
Smoker No Yes Ex-smoker	59 14 5	75.6 18.0 6.4
IgG Chlamydia Negative Positive	68 11	86.1 13.9
Tubal patency HyFoSy Bilateral tubal occlusion Unilateral tubal occlusion Bilateral tubal patency	5 15 59	6.3 19.0 74.7
VAS HyFoSy intraprocedural, Mean (SD)	5.2 (2.4)	-
VAS HyFoSy post-procedural, Mean (SD)	2.6 (2.5)	-
Endometriosis No Yes	76 3	96.2 3.8
Infertility time (months), Mean (SD)^1^	26.4 (15.6)	-
AMH (ng/mL) < 0,6 0,6 - 1,1 > 1,1	3 6 68	3.9 7.8 88.3
Ovarian pathology No Yes^2^	74 5	93.7 6.3
Pelvic inflammatory disease history No Yes	78 1	98.7 1.3
Antral follicle count < 10 10-20 > 20	10 28 41	12.7 35.4 51.9
Soldo programme No Yes	24 55	30.3 69.7

AMH: anti-Müllerian hormone, BMI: body mass index, SD: Standard Deviation, VAS: visual analogue scale.

1 Only for patients not in the SOLDO programme.

2 Type of ovarian pathology: Endometrioma (3), Cyst (2).

Soldo: Soldo in Catalonia: Access to ART for women without a male partner

In the bivariate analysis, the two groups analysed were comparable in terms of confounding factors. No statistically significant associations were found between tubal patency and the variables under study. *Chlamydia* IgG seropositivity was detected in 25.0% of patients with tubal occlusion versus 10.2% among those with bilateral patency (Table 2).

**Table 2:** Description of the study sample according to whether or not they have HyFoSy tubal patency (2 groups)No tubal patency = Bilateral tubal occlusion (n=5) + Unilateral tubal occlusion (n=15) Tubal patency = No tubal blockade (n=59)

	Tubal patency HyFoSy				
	No (n=20)		Yes (n=59)		p-value
	N	%	N	%	
Age (years), Mean (SD)	34.0 (4.1)	-	33 (3.4)	-	0.319^*^
BMI (kg/m2), Mean (SD)	25.7 (4.4)	-	25.1 (5.1)	-	0.688^*^
Smoker No Yes Ex-Smoker	13 5 2	65.0 25.0 10.0	46 9 3	79.3 15.5 5.2	0.429^**^
IgG Chlamydia Negative Positive	15 5	75.0 25.0	53 6	89.8 10.2	0.098^**^
VAS HyFoSy intraprocedural, Mean (SD)	5.4 (2.9)	-	5.2 (2.2)	-	0.791^***^
VAS HyFoSy post-procedural, Mean (SD)	3.1 (2.7)	-	2.4 (2.4)	-	0.280^***^
Endometriosis No Yes	18 2	90 10	58 1	98.3 1.7	0.156^****^
Infertility time (months)1, Mean (SD)	26.6 (15.5)	-	26.3 (15.9)	-	0.784^***^
AMH (ng/mL) < 0,6 0.6 - 1.1 > 1.1	0 1 18	0.0 5.3 94.7	3 5 50	5.2 8.6 86.2	0.520^**^
Ovarian pathology No Yes^2^	18 2	90.0 10.0	56 3	94.9 5.1	0.596^****^
Pelvic inflammatory disease history No Yes	19 1	95.0 5.0	59 0	100 0.0	0.253^****^
Antral follicle count < 10 10 - 20 > 20	1 7 12	5.0 35.0 60.0	9 21 29	15.3 35.6 49.2	0.453^**^
Soldo Programme No Yes	4 16	20.0 80.0	20 39	33.9 66.1	0.277^****^

AMH: anti-Müllerian hormone, BMI: body mass index, SD: Standard Deviation, VAS: visual analogue scale.

1 Only for patients who do not belong to the SOLDO programme.

2 Type of ovarian pathology: Without tubal patency: Endometrioma (2); With bilateral tubal patency: Endometrioma (1) and Cyst (2).

* T test /

** χ^2^ test /

*** Mann-Whitney test /

**** Fisher’s exact test

Soldo: Soldo in Catalonia: Access to ART for women without a male partner

Among patients with positive *Chlamydia* serology, the rate of tubal occlusion was 45.5%, compared to 22.1% among those with negative serology (Table 3). In the multivariate logistic regression model, this association did not reach statistical significance (OR 2.94, 95% CI [0.79, 11.00]; χ^2^ test, *p*-value 0.098) (Table 3). Testing *Chlamydia* IgG serology showed high specificity (90%) and negative predictive value (78%) for tubal occlusion (Table 3).

**Table 3:** Relationship between HyFoSy tubal occlusion (unilateral or bilateral tubal occlusion) and the presence of *Chlamydia* infection stratified by the median patient age

	Tubal occlusion HyFoSy							
	OR	[IC95%]	p-value	%	Sensitivity	Specificity	PPV	NPV
Total Negative Positive	1 2.94	- [0.79; 11.00]	- 0.098 (χ^2^ test)	22.1 45.5	0.25	0.90	0.45	0.78
< 33 years Negative Positive	1 8.00	- [1.08; 59.16]	- 0.025 (χ^2^ test)	11.1 50.0	0.5	0.89	0.5	0.89
≥ 33 years Negative Positive	1 1.61	- [0.24; 10.90]	- 0.623 (χ^2^ test)	29.3 40.0	0.14	0.91	0.4	0.71
PPV: Positive predictive value; NPV: Negative predictive value IC95%: 95% Confidence Interval								

**Figure 1:**
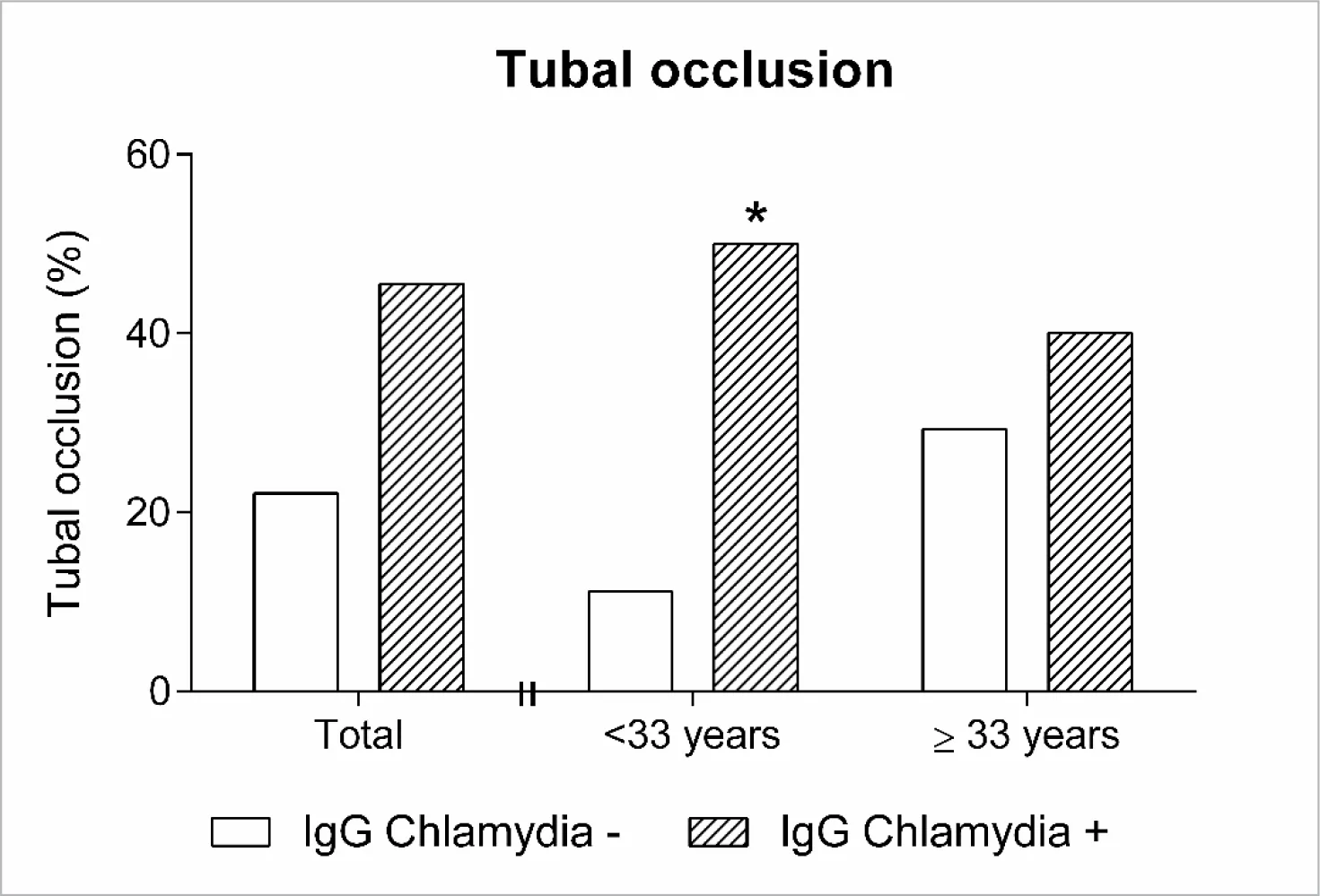
Relationship between HyFoSy tubal occlusion (unilateral or bilateral tubal occlusion) and *Chlamydia* infection history, stratified by patient age. χ^2^ test, * *p*-value < 0.05.

Patients were stratified by the median age (33 years), as age was a confounding variable. Among women under 33 years, the association between *Chlamydia* infection history and tubal occlusion was statistically significant. Tubal occlusion was observed in 50.0% of *Chlamydia*-positive patients, compared to 11.1% in those with negative serology, (OR 8.00, 95% CI [1.08-59.16]; χ^2^ test, *p*-value 0.025) (Table 3, Figure 1). However, this subgroup included a limited number of seropositive cases, resulting in wide confidence intervals that should be interpreted with caution. No statistically significant differences were observed in patients aged 33 years or older (OR 1.61, 95% CI [0.24; 10.90]; χ^2^ test, *p*-value 0.623).

Total sample size: n=79 (n=11 IgG-positive; n=68 IgG-negative).

Sample size <33 years subgroup: n=33 (n=6 IgG-positive; n=27 IgG-negative).

Sample size ≥33 years subgroup: n=46 (n=5 IgG-positive; n=41 IgG-negative).

Note: The number of IgG-positive cases in each subgroup was small, resulting in wide confidence intervals; results should therefore be interpreted with caution.

## DISCUSSION

Our findings indicate a potential association between *Chlamydia* IgG seropositivity and a higher frequency of tubal patency impairment, particularly in women under the age of 33, where the association reached statistical significance.

At present, there are no published studies associating HyCoSy or HyFoSy-non-invasive tests that assess tubal patency-with a history of *Chlamydia trachomatis* infection, one of the main causes of tubal involvement. To the best of our knowledge, this is the first study to explore this relationship using HyFoSy as the diagnostic method. While tubal damage resulting from *Chlamydia* infection is well documented, previous research has primarily relied on laparoscopy or HSG for its detection (Hiroi *et al*., 2007; Morhason-Bello *et al*., 2014).

Several clinical guidelines recommend *Chlamydia trachomatis* IgG serology as a first-line screening tool in infertility evaluations. In cases of seropositivity, diagnostic laparoscopy with chromopertubation is often indicated, bypassing HSG or ultrasound-based methods (Lim *et al*., 2011).

When used alone, *Chlamydia* IgG serology shows a sensitivity between 45-74% and specificity between 83-93% for detecting tubal pathology (Keltz *et al*., 2006; Perquin *et al*., 2007). While the positive predictive value (PPV) may be as high as 94%, the lower negative predictive value (NPV) of 70% limits its usefulness as a standalone tool and supports the need for complementary imaging (Keltz *et al.,* 2006). The diagnostic performance of *Chlamydia* IgG serology improves when combined with HSG. Keltz *et al.* (2006) reported an increase in sensitivity from 78% to 97% when both tests were used, with laparoscopy as the reference standard. Similarly, Broeze *et al.* (2012) reported that the combined use of HSG and serology yielded the highest diagnostic performance. Other studies have reported *Chlamydia* IgG serology to present slightly lower PPVs (32-63%) and higher NPVs (74-90%) when tubal pathology was determined by laparoscopy (van Ess *et al.,* 2019). Additionally, patients with laparoscopically confirmed tubal disease test positive for *Chlamydia* IgG in up to 40% of cases (Keltz *et al.,* 2006; Perquin *et al.,* 2007). IgG seropositivity reflects previous exposure rather than active infection, and does not necessarily correlate with the presence or severity of tubal damage. Therefore, positive serology should be interpreted with caution and in conjunction with complementary imaging or clinical findings.

However, some authors argue that both HSG and *Chlamydia* IgG offer similar diagnostic value, with low predictive power overall, and prefer serology due to its non-invasive nature and lower cost (den Hartog *et al.,* 2008; Perquin *et al.,* 2007). Moreover, even when HSG is normal, seropositive patients still show a 43% chance of having tubal damage confirmed by laparoscopy (Hiroi *et al.,* 2007).

In patients with positive *Chlamydia* IgG, HSG may pose a higher risk of post-procedural complications, mainly infection-estimated at around 10%-while these risks have not been described with HyCoSy in seropositive patients. For seronegative patients, HyCoSy is often recommended as the preferred non-invasive method for evaluating tubal patency (Lim *et al.,* 2011). HyCoSy is better tolerated than HSG, with a lower rate of severe intraprocedural pain (Boned-López *et al.,* 2021), and does not require ionising radiation or iodinated contrast, making it more accessible and better tolerated overall (Ramos *et al*., 2021; 2022; van Rijswijk *et al*., 2018).

Additionally, HyCoSy provides higher-resolution assessment of the uterine cavity and adnexa compared to basic transvaginal ultrasound. When foam is used as the contrast medium-known as HyFoSy-diagnostic accuracy improves significantly, with sensitivity and specificity equal to or greater than those of HyCoSy (Exalto & Emanuel, 2019; Lim *et al*., 2015). In the present study, the mean immediate post-procedural pain score reported for HyFoSy was 2.6 on the VAS scale, consistent with previous findings and supporting its excellent tolerability in routine clinical settings (Boned-López *et al.,* 2021).

The presence of *Chlamydia trachomatis* IgG antibodies has been associated with reduced reproductive outcomes. Seropositive patients have lower rates of spontaneous pregnancy (RR 0.65) and live birth (RR 0.59) compared to seronegative patients (Steiner *et al.,* 2015). Other studies have reported a 33% to 57% reduction in spontaneous conception rates among seropositive women, along with a higher prevalence of tubal pathology on HSG (37.5% *vs*. 10.1%) and higher rates of tubal damage confirmed by laparoscopy (86% *vs*. 49%) (Coppus *et al.,* 2011).

*Chlamydia* IgG seropositivity has also been linked to poorer outcomes in in vitro fertilization (IVF) and higher miscarriage rates, although findings are not consistent across studies (Keltz *et al*., 2006; 2013). In clinical practice, the coexistence of tubal pathology on HSG and positive *Chlamydia* serology is considered a sufficient indication to proceed directly to IVF, without requiring laparoscopic confirmation-except in cases with hydrosalpinx, where salpingectomy may be indicated (Lim *et al*., 2011).

The study population was homogeneous, comprising women of reproductive age with preserved ovarian reserve and favourable baseline fertility indicators. A higher incidence of tubal factor infertility was observed among patients with positive *Chlamydia trachomatis* IgG serology, reinforcing the association between prior infection and tubal damage. This association was particularly evident in younger patients, consistent with previous studies linking *Chlamydia* primoinfection before the age of 20 to an increased risk of tubal pathology and infertility (Hoenderboom *et al.,* 2019).

Given its accuracy, non-invasiveness, and good tolerability, HyFoSy is increasingly being adopted as the first-line modality in the evaluation of tubal patency during infertility workups. Its widespread use may facilitate earlier detection of tubal involvement, especially in high-risk groups such as women with a history of *Chlamydia* infection.

This study presents several strengths. All patients underwent standardized diagnostic procedures, including blinded evaluation of tubal patency by experienced specialists, which reduces the risk of observer bias. The use of *Chlamydia trachomatis* IgG serology as a screening tool offers a simple, inexpensive, and non-invasive method that can be readily implemented in routine infertility assessments. Moreover, this is the first study to explore the association between *Chlamydia* IgG seropositivity and tubal patency as assessed by HyFoSy-a non-invasive technique that is increasingly being adopted as the first-line method for evaluating tubal status-thus contributing novel insights to the field of reproductive medicine.

The main limitations of this pilot study include its retrospective design and the potential for selection bias inherent to single-centre recruitment. These factors, together with the relatively small sample size, may restrict the statistical power and limit the generalizability of the findings to broader populations and clinical settings. Another important limitation is the absence of confirmatory diagnosis through invasive methods such as laparoscopy, which remains the gold standard for detecting tubal pathology. Furthermore, tubal patency was assessed exclusively using HyFoSy, whose sensitivity and specificity, although high, may be lower than those of laparoscopy with chromopertubation, potentially influencing the observed results. These limitations should be considered when interpreting the results, which require validation through larger prospective multicentric studies.

Although adhesions secondary to Chlamydia infection can affect fertility, this parameter was not evaluated in the present study. HyFoSy does not allow direct visualization of peritoneal adhesions, and potential indirect signs were not systematically collected. This constitutes an additional methodological limitation. While the HyFoSy process does not specifically address this issue, the test can still be useful in identifying it in certain cases.

Despite growing evidence supporting the utility of *Chlamydia trachomatis* IgG serology in infertility screening, its routine implementation remains limited, possibly due to the stigma associated with sexually transmitted infections (Keltz *et al*., 2006). The present findings support the inclusion of *Chlamydia* IgG testing in the infertility work-up of young women, particularly those at higher risk of prior asymptomatic infection. However, it is important to note that serological testing cannot replace imaging-based assessment of tubal patency. Its predictive performance for detecting clinically significant tubal pathology remains modest, and its clinical impact in isolation is therefore limited. In addition, the introduction of routine serology implies additional cost and resource use, which may restrict its application to selected patient populations where risk of prior infection is high. Consequently, *Chlamydia* serology should be viewed as a complementary rather than a primary screening tool within infertility evaluation protocols.

HyFoSy has become a preferred first-line diagnostic tool for assessing tubal patency, owing to its effectiveness, ease of performance, and favourable tolerability profile (Bohîlţea *et al*., 2022; Grigovich *et al*., 2021; Kowalczyk *et al*., 2021; Rodríguez Pérez *et al*., 2022; Rajesh *et al*., 2016). Its application in clinical practice spans over a decade, with multiple studies validating its diagnostic value (Emanuel *et al*., 2009). When combined with three-dimensional transvaginal ultrasound, HyFoSy enables comprehensive evaluation of uterine, ovarian, and tubal structures in a single session. This integrated approach-commonly referred to as the “one-stop fertility scan”-is minimally invasive, safe, time-efficient, and facilitates earlier initiation of assisted reproductive treatments, potentially shortening time to pregnancy (Kowalczyk *et al*., 2021; Levaillant *et al*., 2019; Zajicek *et al*., 2022). Notably, the addition of 3D/4D imaging does not appear to significantly enhance diagnostic accuracy (Alcázar *et al*., 2022; Exalto & Emanuel, 2019).

While several studies have demonstrated an association between *Chlamydia trachomatis* infection and tubal factor infertility using HSG with iodinated contrast, there is currently a lack of evidence evaluating this relationship using foam-based ultrasound techniques such as HyFoSy. Given the increasing use of HyFoSy as a first-line diagnostic modality in infertility workups, establishing its association with serological evidence of prior *Chlamydia* infection may offer valuable insiamyghts for improving clinical decision-making.

The present findings may suggest a possible change in clinical considerations regarding the use of artificial insemination by husband (AIH) in women with bilateral tubal patency confirmed by HyFoSy but who test positive for *Chlamydia* IgG. In such cases, earlier consideration of IVF could be explored, as positive serology may reflect underlying subclinical tubal dysfunction not detected by imaging alone. This hypothesis remains speculative and warrants further investigation in prospective studies. We can also consider offering laparoscopy as a treatment for tubal occlusion, as it is an alternative that has been described with good pregnancy outcomes (Watrelot & Chauvin, 2011; Watrelot & Guan, 2021).

In conclusion, this study suggests a potential relevance of *Chlamydia trachomatis* IgG seropositivity and impaired tubal patency, as a complementary tool in the assessment of tubal factor infertility, particularly in younger women. Integrating serological findings with non-invasive imaging techniques like HyFoSy could contribute to optimize reproductive planning and treatment strategies in clinical practice. However, the retrospective nature of this study precludes establishing causality, and further prospective research is required to confirm these findings.

## CONCLUSIONS

A potential association was observed between *Chlamydia trachomatis* IgG seropositivity and impaired tubal patency as assessed by HyFoSy. This association reached statistical significance in women under 33 years of age, suggesting that prior *Chlamydia* infection may be associated with a higher risk of tubal occlusion in younger patients. These findings would support the role of *Chlamydia* infection as a contributing factor to tubal infertility. Further studies are needed to confirm these results and to evaluate the impact of past *Chlamydia* infection on the selection and outcomes of assisted reproductive techniques.
